# Orange juice with a high-fat meal prolongs postprandial lipemia in apparently healthy overweight/obese women

**DOI:** 10.1590/2359-3997000000229

**Published:** 2016-11-07

**Authors:** Raquel Cristina L. A. Coelho, Helen Hermana M. Hermsdorff, Renata S. Gomide, Raquel Duarte M. Alves, Josefina Bressan

**Affiliations:** 1 Departamento de Nutrição e Saúde Universidade Federal de Viçosa Viçosa MG Brasil Departamento de Nutrição e Saúde, Universidade Federal de Viçosa (UFV), Viçosa, MG, Brasil

**Keywords:** Orange juice, high-fat meal, postprandial lipemia, obesity

## Abstract

**Objective:**

We investigated the postprandial response of lipid markers to a high-fat meal (HFM) with two different beverages in apparently healthy normal-weight and overweight/obese women.

**Subjects and methods:**

This crossover, randomized study enrolled 36 women, of whom 21 had normal weight (body mass index [BMI] 22 ± 1.8 kg/m^2^) and 15 had overweight/obesity (BMI 31 ± 3.7 kg/m^2^). In two different test days, the participants ingested a HFM (37% of energy as saturated fat) with 500 mL of water (HFM-W) or 500 mL of orange juice (HFM-OJ). Blood samples were collected at baseline (12-hour fasting), and at 2, 3, and 5 hours postprandial. The analysis included fasting and postprandial total cholesterol, HDL-c, LDL-c, triglycerides (TG), uric acid, and complement C3. Brazilian Clinical Trials Registry (ReBEC); Primary Identification Number: RBR-2h3wjn (www.ensaiosclinicos.gov.br).

**Results:**

TG levels increased at 3 hours with HFM-OJ in normal-weight women (p = 0.01) and returned to normal levels at 5h. TG increased at 3 hours with HFM-W (p = 0.01) and HFM-OJ (p = 0.02), and remained high at 5 hours (p = 0.03) in overweight/obese women. Complement C3 remained unchanged, but showed different responses between meals (p = 0.01 for positive incremental area under the curve [piAUC] HFM-OJ vs. HFM-W, respectively).

**Conclusions:**

In apparently healthy overweight/obese women compared with normal-weight ones, the concomitant intake of orange juice with a HFM prolonged postprandial lipemia but had no effect on postprandial complement C3 concentrations.

## INTRODUCTION

Postprandial lipemia (PPL) refers to the dynamic changes in serum lipids and lipoproteins that occur after a fat load or meal. These responses are reflected mainly in changes in plasma triglycerides (TG). TG-rich lipoproteins and their remnants are known as risk predictors of coronary heart disease ( 1 - 2 ). Indeed, PPL has gained interest since recent reports have demonstrated that increases in postprandial TG levels are possibly even stronger independent predictors of cardiovascular diseases than fasting TG ( 3 ). Moreover, complement C3 has been positively associated with obesity, insulin resistance, metabolic syndrome features, and fasting and postprandial TG ( 4 ).

In this sense, the Western dietary pattern, characterized by a consumption of high-energy density diets and refined foods, contributes to a positive energy balance leading to weight gain and obesity and may trigger low-grade systemic inflammation and metabolic syndrome abnormalities ( 5 , 6 ). Western individuals remain in a postprandial state for most of the day ( 7 ). Consequently, repeated acute dietary stressors induced by a high-fat meal (HFM) could trigger a large increase of most risk factors for cardiovascular diseases related to obesity, such as increased circulating cholesterol, TG, and glucose ( 8 ). In turn, fruit intake has been associated with an improvement in lipid profile and reduction in concentrations of inflammatory and oxidative stress markers ( 9 , 10 ). We have previously published a review of studies assessing the anti-inflammatory properties of orange juice (OJ), which appears to mediate the plasma levels and gene expression of factors involved in metabolic and inflammatory responses in postprandial and chronic (≥ 7 consecutive days) periods ( 11 ).

In the present study, we investigated the postprandial response of lipid markers to a HFM with two different beverages (water and OJ) in apparently healthy normal-weight and overweight/obese women.

## SUBJECTS AND METHODS

### Subjects

Recruitment was conducted through the university website, posters, and active search in clinical and medical service centers. In total, 74 women were recruited and 45 were enrolled in the study. Six women failed to complete the study claiming lack of time and three presented technical problems in blood collection. The participants comprised normal-weight (n = 21) and overweight/obese (n = 15) women. They were apparently healthy with no recent acute or chronic inflammatory diseases and/or use of anti-inflammatory or immunosuppressive drugs and steroids. They were non-smokers and, to be enrolled, could not be pregnant or nursing. Subjects were excluded if they had any past or present cardiovascular disease, diagnosed diabetes or any inflammatory condition, or used medications known to affect the study outcomes. Approval for the study was obtained from the Ethics Committee for Human Research at Universidade Federal de Viçosa (Of. Ref. Nº 184/2011) and all procedures involving human subjects complied with the Declaration of Helsinki, as revised in 2000. All participants signed a written informed consent form. The study is registered at the Brazilian Clinical Trials Registry (ReBEC; www.ensaiosclinicos.gov.br) with the primary identification number RBR-2h3wjn.

### Study design

The dietary intervention followed a randomized crossover design, with a washout period of at least 7 days between the test days. Two days prior to each test day, the subjects followed a low-antioxidant diet (washout) by avoiding olive and fish oils, fresh fruits and vegetables, tea, coffee, fruit juices, and wine. The subjects were then randomly assigned to either a HFM plus 500 mL of water (HFM-W) or a HFM plus 500 mL OJ (HFM-OJ) group. On the morning of each test day, the participants arrived at the laboratory at 7:00-7:30 am. Body weight, height, and blood pressure were measured, and a fasting blood sample was taken before the test meal. Body weight and height were measured using standard procedures according to previously described protocols ( 12 ). Body mass index (BMI) was calculated using the equation: BMI = weight (kg)/height^2 ^(m^2^). The percentage of body fat was estimated by bioelectrical impedance analysis (Biodynamics 310e, Chicago, USA) using standard protocols ( 12 ). Blood pressure was measured using a standard mercury sphygmomanometer, with the patient in the seated position.

The test meals were served at 7:30-8:00 am and consumed within 30 min. Postprandial blood samples were obtained at 2, 3, and 5 hours after the beginning of the test meals. The subjects remained in the laboratory and were not allowed to consume any additional foods or beverages, except for water.

### Composition of the meals

The HFM consisted of two muffins with bacon and cheese (90g each), providing 1010 kcal, with 78% of energy as fat (37% as saturated fat), 16% as carbohydrates, and 6% as protein. The muffins were accompanied by 500 mL of water (HFM-W) or OJ (HFM-OJ) in different test meal days. Concentrated integral sugar-free OJ (100% OJ) was provided by Fast Fruit^®^ in 1-liter packages; an amount of 500 mL provides 215 kcal from 50g of carbohydrates (information provided by the manufacturer). The juice package was opened at the time of the consumption.

### Assessment of metabolic markers

Fasting and postprandial blood samples were collected in EDTA tubes using a 21G butterfly needle and were centrifuged immediately at 2,200 x g at 5ºC for 15 min. The plasma was then separated and stored at -80ºC. The analyses were performed in the semiautomatic analyzer BS200 (Mindray, Nanshan, China). Plasma concentrations of TG, total cholesterol, HDL-c, LDL-c, uric acid, and glucose were measured using colorimetric enzymatic commercial kits (Bioclin, Belo Horizonte, Brazil). Plasma concentrations of complement C3 were measured by turbidimetric methods using commercially available kits (Bioclin, Belo Horizonte, Brazil).

### Statistical analysis

The results are presented as mean ± standard deviation (SD). Age, BMI, body weight and composition, and plasma baseline metabolic biomarkers were compared between groups using Student’s *t* test or Mann-Whitney test, as appropriate. As the groups (normal-weight and overweight/obese) differed in age, the analyses were adjusted for this variable (Tukey-Kramer test). A mixed model using a three-way repeated-measures ANOVA was applied to test the differences between test meals throughout the test day for postprandial metabolic and inflammatory variables with test meals, groups, and time as repeated factors. *Post hoc* testing was performed using the Tukey-Kramer test. The statistical analyses were performed using the SAS statistical package (version 9.2; SAS Institute Inc, Cary, NC, USA). The rejection level of significance used was 5%. The incremental area under the curve (piAUC) was calculated using GraphPad Prism (Version 5; GraphPad Software Inc., USA). Power analysis, calculated with the analyst procedure of SAS, indicated that a sample of 15 subjects per group would allow the detection of a treatment effect accounting for 5% of the within-subject variance in TG with more than 99% of power at the 5% level of probability.

## RESULTS

### Baseline

A total of 36 women completed the study and served as controls for themselves. Baseline values of weight, BMI, body fat, glucose, HDL-c, and uric acid differed between groups (normal weight vs. overweight/obese), as presented in Table 1 .

**Table 1:** Baseline characteristics of the participants

	Normal-weight women	Overweight/Obese women	P value
Participants (n)	21	15	
Age (y)	24 ± 4	31 ± 8	0.022
Weight (kg)	58 ± 5	81.4 ± 13	< 0.001
BMI (kg/m^2^)	22 ± 1.8	31.1 ± 3.7	< 0.001
Body fat (%)	25.8 ± 3.2	37 ± 3.2	< 0.001
SBP (mmHg)	103.3 ± 7.2	110.6 ± 8.8	0.200
DBP (mmHg)	64.8 ± 6.7	69.4 ± 8.7	0.103
Glucose (mg/dL)	88.2 ± 6.5	97.9 ± 7	0.004
TC (mg/dL)	168.5 ± 31.6	168.21 ± 26.4	0.793
HDL-c (mg/dL)	67.2 ± 17.2	50.8 ± 7.1	0.005
LDL-c (mg/dL)	81.4 ± 24	87 ± 16.5	0.672
TG (mg/dL)	96.8 ± 32.8	136.3 ± 65.8	0.176
Uric acid (mg/dL)	3.7 ± 0.7	4.4 ± 0.6	0.004
C3 (mg/dL)	137.5 ± 29.3	142.9 ± 25.9	0.436

Values are expressed as mean ± standard deviation (SD).

BMI: body mass index; MBR: basal metabolic rate; SBP: systolic blood pressure; DBP: diastolic blood pressure; TC: total cholesterol; TG: triglycerides.

P values for comparisons between groups using Student’s *t* test or Mann-Whitney test. The results remained after adjustment by age (Tukey-Kramer test).

### Metabolic postprandial response

**Figure 1:**
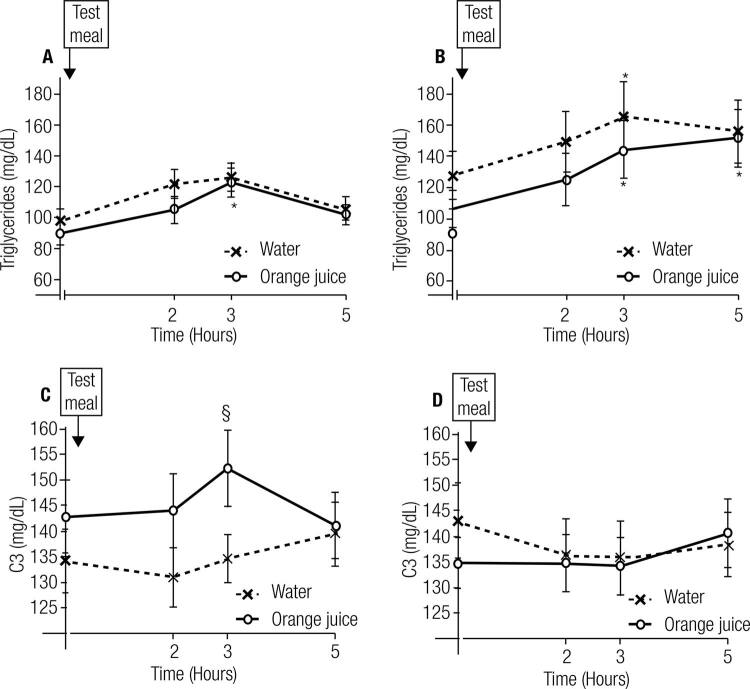
Line plots showing the changes (as mean ± standard error) in plasma triglycerides and C3 after a high-fat meal plus water (HFM-W) and a high-fat meal plus orange juice (HFM-OJ) in normal-weight (A and C, respectively) and overweight (B and D, respectively) participants. Mixed model using three-way repeated-measures ANOVA followed by Tukey-Kramer *post hoc* analysis: * p < 0.05; § p < 0.01. Difference between beverages at a single point.

After consumption of the HFM-W meal, TG levels tended to increase in normal-weight volunteers at 3 hours postprandial relative to fasting (p = 0.07). However, this increase at the third hour was only significant when these participants consumed the HFM-OJ meal (p = 0.01). At 5 hours, there was no difference between baseline and postprandial TG in normal-weight women (p = 0.99). Overweight/obese women presented increased TG in relation to fasting at 3 hours postprandial both after the HFM-W (p = 0.01) and HFM-OJ (p = 0.02) meals. Furthermore, the increase in TG compared with fasting remained at 5 hours after consumption of the HFM-OJ meal in overweight/obese volunteers (p = 0.03) ( Figure 1 ).

At 5 hours postprandial, there was a trend towards a difference in TG levels between the groups of normal-weight and overweight/obese volunteers (p = 0.06). Complement C3 presented a significantly higher piAUC (p = 0.03) in the normal-weight group.

Total cholesterol, HDL-c, and LDL-c did not vary statistically over time or between diets.

## DISCUSSION

In this study, PPL occurred 3 hours after the ingestion of a HFM. Studies have shown that circulating TG presents a pronounced increase ( *i.e.* , PPL) 1 hour after consumption of a typical fat-containing meal (30–60g of fat) and may remain high for 5–8 hours ( 13 , 14 ). Recent epidemiological studies have clearly evidenced the predictive relationship between the extent of postprandial hypertriglyceridemia and a relative risk of cardiovascular events ( 15 , 16 ). This finding is relevant concerning the analyses of lipids as biomarkers in chronic diseases.

The relevant finding of this study was that overweight/obese women consuming a HFM-OJ meal had a prolonged TG increase, with higher TG levels at 5 hours postprandial. Peairs and cols. reported increased postprandial TG in obese patients ( 17 ). The first point of the discussion is the difference in PPL between normal-weight and overweight/obese women. Normal-weight women presented a TG increase only after the HFM-OJ meal, while overweight/obese volunteers had a TG increase after both meals. In fact, the amplitude and duration of the PPL are related to the meal composition and the physiopathological condition of the subjects, including obesity ( 18 , 19 ). PPL in overweight/obese women disclosed a lipid intolerance state that could not be detected in fasting. In obese humans, fasting plasma lipids can be normal but postprandial lipid metabolism is abnormal with an accumulation of TG-rich remnant lipoproteins. In addition, the catabolism of their chylomicrons (CM) remnants was markedly decreased when compared with lean women ( 20 ). The decreased clearance of CM remnants in overweight/obese subjects may be explained by a competition between these remnants and the increased hepatic production of VLDL for clearance by low-density lipoprotein receptors.

The second point of the discussion is the role of OJ on PPL in normal-weight and overweight/obese women. Studies about the effect of fruit-derived antioxidant and fructose intake on fatty meal-induced metabolic changes have reported contradictory results. We observed an enhanced PPL when OJ was added to the HFM. Similar to our findings, Stanhope and cols. observed that diets rich in highly digestible carbohydrates can lead to higher levels of fasting plasma TG as a result of hepatic VLDL and CM remnant accumulation due to altered lipoprotein secretion and/or clearance ( 21 ). Cerletti and cols. found that the concomitant intake of OJ resulted in a reduced increase in plasma TG and reduction in total cholesterol ( 22 ). These findings altogether indicate that postprandial metabolism resulting from the digestion and absorption of available nutrients is a highly complex process involving numerous potential interactions. In addition, other studies have shown that the amount or type of carbohydrate in a meal alter the postprandial lipid metabolism ( 23 ). However, data obtained after addition of glucose (50 to 100g) to a HFM have not provided reproducible findings in healthy subjects ( 24 ). Thus, despite epidemiological data suggesting an inverse association between citrus fruits intake and cardiovascular disease risk, our understanding of the mechanisms by which flavonones potentially reduce this risk remains unclear.

As a limitation of the study, the total energy intake in the meal with OJ was 215 kcal higher than that in the meal with water. The addition of OJ increased the amount of carbohydrates in the meal (40.7g for HFM-W vs. 91g for HFM-OJ), without contributing to fat content. Given its fructose content, OJ may have altered the PPL by increasing hepatic fat synthesis ( 25 ). However, Stookey and cols. have shown that the addition of OJ to a meal with 12g of fat limited fat oxidation in the postprandial period ( 26 ). These results suggest that reduced fat oxidation might mediate the effects of caloric beverages on weight gain, independent of energy excess. In adults, reduced fat oxidation predicts weight gain, independent of metabolic rate ( 27 ). Another point is the 48-hour washout diet applied before the test meal days. This low-antioxidant diet aimed to minimize the variability of the biochemical and inflammatory markers analyzed, which was important in view of the physiological approach in the current study. However, this diet could hamper the application of the results in free-living conditions.

Regarding complement C3, some studies have shown a correlation between fasting C3 and TG ( 28 , 29 ). However, we did not find correlations between fasting C3 and metabolic syndrome parameters, probably because of the small number of individuals assessed. Furthermore, Halkes and cols. have found plasma C3 levels significantly higher than fasting at 2, 4, and 6 hours after fat ingestion (50g/m^2 ^of fat) by normolipidemic subjects with coronary artery disease and healthy controls ( 30 ). We did not find a significant postprandial difference in plasma C3. Possible causes for this negative outcome are the different populations assessed, different fat overload, and the addition of OJ. Charlesworth and cols. also found no significant postprandial changes in C3 after a mixed meal, although their study lacked information about the amount of fat ( 31 ). Van Oostrom and cols., while studying the relationship of PPL with meal composition in healthy subjects, observed that the addition of glucose to a fat overload decreased PPL and prevented a fat-specific increase in C3 ( 32 ). For Schär and cols., OJ or hesperidin supplement did not acutely affect cardiovascular risk biomarkers ( 33 ). Because of existing controversial results concerning postprandial response of complement C3 to meal composition, more studies are necessary to investigate the biochemical and dietary factors related to variations in circulating C3 in healthy and obese people.

In conclusion, our study has shown that overweight/obese women have enhanced PPL when compared with normal-weight women, and that the increase in TG was more prolonged when these overweight/obese volunteers consumed a HFM with OJ. Since the findings of our present study and previously published ones are still controversial, more studies are necessary to clarify the role of OJ or citrus juice intake in the lipid metabolism and in the prevention of chronic diseases.

## References

[1] Kolovou G, Ooi TC. Postprandial lipaemia and vascular disease. Curr Opin Cardiol. 2013;28(4):446-51. 10.1097/HCO.0b013e3283606971 23591556

[2] Sarwar N, Sandhu MS, Ricketts SL, Butterworth AS, Di Angelantonio E, Boekholdt SM, et al. Triglyceride-mediated pathways and coronary disease: collaborative analysis of 101 studies. Lancet. 2010;375(9726):1634-9. 10.1016/S0140-6736(10)60545-4 PMC2867029 20452521

[3] Nordestgaard BG, Benn M, Schnohr P, Tybjaerg-Hansen A. Nonfasting triglycerides and risk of myocardial infarction, ischemic heart disease, and death in men and women. JAMA. 2007;298(3):299-308. 10.1001/jama.298.3.299 17635890

[4] Klop B, Proctor SD, Mamo JC, Botham KM, Castro Cabezas M. Understanding postprandial inflammation and its relationship to lifestyle behaviour and metabolic diseases. Int J Vasc Med. 2012:947417. 10.1155/2012/947417 PMC3179890 21961070

[5] Bressan J, Hermsdorff HH, Zulet MA, Martinez JA. Hormonal and inflammatory impact of different dietetic composition: emphasis on dietary patterns and specific dietary factors. Arq Bras Endocrinol Metabol. 2009;53(5):572-81. 10.1590/s0004-27302009000500010 19768248

[6] Van Oostrom AJ, Alipour A, Plokker TW, Sniderman AD, Cabezas MC. The metabolic syndrome in relation to complement component 3 and postprandial lipemia in patients from an outpatient lipid clinic and healthy volunteers. Atherosclerosis. 2007;190(1):167-73. 10.1016/j.atherosclerosis.2006.01.009 16488421

[7] Nakajima K, Nakano T, Tokita Y, Nagamine T, Inazu A, Kobayashi J, et al. Postprandial lipoprotein metabolism: VLDL vs chylomicrons. Clin Chim Acta. 2011;412(15-16):1306-18. 10.1016/j.cca.2011.04.018 PMC3265327 21531214

[8] Sharrett AR, Heiss G, Chambless LE, Boerwinkle E, Coady SA, Folsom AR, et al. Metabolic and lifestyle determinants of postprandial lipemia differ from those of fasting triglycerides: The Atherosclerosis Risk In Communities (ARIC) study. Arterioscler Thromb Vasc Biol. 2001;21(2):275-81. 10.1161/01.atv.21.2.275 11156865

[9] Hermsdorff HH, Zulet MA, Puchau B, Martinez JA. Fruit and vegetable consumption and proinflammatory gene expression from peripheral blood mononuclear cells in young adults: a translational study. Nutr Metab (Lond). 2010;7:42. 10.1186/1743-7075-7-42 PMC2882916 20465828

[10] Cocate PG, Natali AJ, Oliveira AD, Longo GZ, Alfenas Rde C, Peluzio Mdo C, et al. Fruit and vegetable intake and related nutrients are associated with oxidative stress markers in middle-aged men. Nutrition. 2014;30(6):660-5. 10.1016/j.nut.2013.10.015 24631385

[11] Coelho RC, Hermsdorff HH, Bressan J. Anti-inflammatory properties of orange juice: possible favorable molecular and metabolic effects. Plant Foods Hum Nutr. 2013;68(1):1-10. 10.1007/s11130-013-0343-3 23417730

[12] de Oliveira FC, Alves RD, Zuconi CP, Ribeiro AQ, Bressan J. Agreement between different methods and predictive equations for resting energy expenditure in overweight and obese Brazilian men. J Acad Nutr Diet. 2012;112(9):1415-20. 10.1016/j.jand.2012.06.004 22939443

[13] Lairon D, Defoort C (2011) Effects of nutrients on postprandial lipemia. Curr Vasc Pharmacol. 2011;9(3):309-12. 10.2174/157016111795495576 21314626

[14] Wierzbicki AS, Clarke RE, Viljoen A, Mikhailidis DP. Triglycerides: a case for treatment? Curr Opin Cardiol. 2012;27(4):398-404. 10.1097/HCO.0b013e328353adc1 22565137

[15] Goldberg IJ, Eckel RH, McPherson R. Triglycerides and heart disease: still a hypothesis? Arterioscler Thromb Vasc Biol. 2011;31(8):1716-25. 10.1161/ATVBAHA.111.226100 PMC3141088 21527746

[16] Freiberg JJ, Tybjaerg-Hansen A, Jensen JS, Nordestgaard BG. Nonfasting triglycerides and risk of ischemic stroke in the general population. JAMA. 2008;300(18):2142-52. 10.1001/jama.2008.621 19001625

[17] Peairs AD, Rankin JW, Lee YW. Effects of acute ingestion of different fats on oxidative stress and inflammation in overweight and obese adults. Nutr J. 2011;10:122. 10.1186/1475-2891-10-122 PMC3225315 22059644

[18] Herieka M, Erridge C. High-fat meal induced postprandial inflammation. Mol Nutr Food Res. 2014;58(1):136-46. 10.1002/mnfr.201300104 23847095

[19] Tam CS, Viardot A, Clement K, Tordjman J, Tonks K, Greenfield JR, et al. Short-term overfeeding may induce peripheral insulin resistance without altering subcutaneous adipose tissue macrophages in humans. Diabetes. 2010;59(9):2164-70. 10.2337/db10-0162 PMC2927938 20547978

[20] Lopez-Miranda J, Williams C, Lairon D. Dietary, physiological, genetic and pathological influences on postprandial lipid metabolism. Br J Nutr. 2007;98(3):458-73. 10.1017/S000711450774268X 17705891

[21] Stanhope KL, Schwarz JM, Keim NL, Griffen SC, Bremer AA, Graham JL, et al. Consuming fructose-sweetened, not glucose-sweetened, beverages increases visceral adiposity and lipids and decreases insulin sensitivity in overweight/obese humans. J Clin Invest. 2009;119(5):1322-34. 10.1172/JCI37385 PMC2673878 19381015

[22] Cerletti C, Gianfagna F, Tamburrelli C, De Curtis A, D’Imperio M, Coletta W, et al. Orange juice intake during a fatty meal consumption reduces the post-prandial low grade inflammatory response in healthy subjects. Thromb Res. 2015;135(2):255-9. 10.1016/j.thromres.2014.11.038 25550188

[23] Parks EJ, Krauss RM, Christiansen MP, Neese RA, Hellerstein MK. Effects of a low-fat, high-carbohydrate diet on VLDL-triglyceride assembly, production, and clearance. J Clin Invest. 1999;104(8):1087-96. 10.1172/JCI6572 PMC408572 10525047

[24] Roche HM, Gibney MJ. Long-chain n-3 polyunsaturated fatty acids and triacylglycerol metabolism in the postprandial state. Lipids. 1999;34 Suppl:S259-65. 10.1007/BF02562313 10419173

[25] Lairon D. Macronutrient intake and modulation on chylomicron production and clearance. Atheroscler Suppl. 2008;9(2):45-8. 10.1016/j.atherosclerosissup.2008.05.006 18595783

[26] Stookey JD, Hamer J, Espinoza G, Higa A, Ng V, Tinajero-Deck L, et al. Orange juice limits postprandial fat oxidation after breakfast in normal-weight adolescents and adults. Adv Nutr. 2012;3(4):629S-635S. 10.3945/an.112.001990 PMC3649737 22798004

[27] Melanson EL, MacLean PS, Hill JO. Exercise improves fat metabolism in muscle but does not increase 24-h fat oxidation. Exerc Sport Sci Rev. 2009;37:93-101. 10.1097/JES.0b013e31819c2f0b PMC2885974 19305201

[28] Muscari A, Massarelli G, Bastagli L, Poggiopollini G, Tomassetti V, Drago G, et al. Relationship of serum C3 to fasting insulin, risk factors and previous ischaemic events in middle-aged men. Eur Heart J. 2000;21(13):1081-90. 10.1053/euhj.1999.2013 10843826

[29] Hermsdorff HHM, Puchau B, Zulet MA, Martínez JA. Association of body fat distribution with proinflammatory gene expression in peripheral blood mononuclear cells from young adult subjects. OMICS. 2010;14(3):297-307. 10.1089/omi.2009.0125 20450441

[30] Halkes CJ, van Dijk H, de Jaegere PP, Plokker HW, van Der Helm Y, Erkelens DW, et al. Postprandial increase of complement component 3 in normolipidemic patients with coronary artery disease: effects of expanded-dose simvastatin. Arterioscler Thromb Vasc Biol. 2001;21(9):1526-30. 10.1161/hq0901.095276 11557683

[31] Charlesworth JA, Peake PW, Campbell LV, Pussel BA, O’Grady S, Tzilopoulos T. The influence of oral lipid loads on acylation stimulating protein (ASP) in healthy volunteers. Int J Obes Relat Metab Disord. 1998;22(11):1096-102. 10.1038/sj.ijo.0800733 9822948

[32] Van Oostrom AJ, Dijk HV, Verseyden C, Sniderman AD, Cianflone K, Rabelink T, et al. Addition of glucose to an oral fat load reduces postprandial free fatty acids and prevents the postprandial increase in complement component 3. Am J Clin Nutr. 2004;79(3):510-5. 10.1093/ajcn/79.3.510 14985229

[33] Schär MY, Curtis PJ, Hazim S, Ostertag LM, Kay CD, Potter JF, et al. Orange juice derived flavanone and phenolic metabolites do not acutely affect cardiovascular risk biomarkers: a randomized, placebo controlled, crossover trial in men at moderate risk of cardiovascular disease. Am J Clin Nutr. 2015;101(5):931-8. 10.3945/ajcn.114.104364 PMC4409690 25788001

